# Evidence that PP2A activity is dispensable for spindle assembly checkpoint-dependent control of Cdk1

**DOI:** 10.18632/oncotarget.23329

**Published:** 2017-12-16

**Authors:** Nando Cervone, Rosa Della Monica, Angela Flavia Serpico, Cinzia Vetrei, Mario Scaraglio, Roberta Visconti, Domenico Grieco

**Affiliations:** ^1^ CEINGE Biotecnologie Avanzate, 80145 Naples, Italy; ^2^ DMMBM, University of Naples “Federico II”, 80131 Naples, Italy; ^3^ IEOS, CNR, 80131 Naples, Italy

**Keywords:** protein phosphatase, PP2A, PP1, spindle assembly checkpoint, SAC

## Abstract

Progression through mitosis, the cell cycle phase deputed to segregate replicated chromosomes, is granted by a protein phosphorylation wave that follows an activation-inactivation cycle of cyclin B-dependent kinase (Cdk) 1, the major mitosis-promoting enzyme. To ensure correct chromosome segregation, the safeguard mechanism spindle assembly checkpoint (SAC) delays Cdk1 inactivation by preventing cyclin B degradation until mitotic spindle assembly. At the end of mitosis, reversal of bulk mitotic protein phosphorylation, downstream Cdk1 inactivation, is required to complete mitosis and crucially relies on the activity of major protein phosphatases like PP2A. A role for PP2A, however, has also been suggested in spindle assembly and SAC-dependent control of Cdk1. Indeed, PP2A was found in complex with SAC proteins while small interfering RNAs (siRNAs)-mediated downregulation of PP2A holoenzyme components affected mitosis completion in mammalian cells. However, whether the SAC-dependent control of Cdk1 required the catalytic activity of PP2A has never been directly assessed. Here, using two PP2A inhibitors, okadaic acid and LB-100, we provide evidence that PP2A activity is dispensable for SAC control of Cdk1 in human cells.

## INTRODUCTION

The PP2A holoenzyme is composed of one catalytic (C) subunit, one scaffold (A) subunit and one of many regulatory (B) subunits that provide substrate and subcellular localization specificity. PP2A-B subunit consists of four distinct subfamilies B55, B56, B” and B”’, with various isoforms for each subfamily [1, 2]. Different PP2A holoenzymes, containing different B subunits, have been involved in mitotic control [1]. It is well established that activity of PP2A-B55 is needed at the end of mitosis for crucial dephosphorylations required for correct execution of late mitotic events like cytokinesis, nuclear envelope reformation, etc., while the phosphatase is actively inhibited during mitosis onset through a recently identified pathway [3, 4]. During mitosis onset, Greatwall kinase (Gwl), another important mitotic kinase activated by Cdk1, phosphorylated two closely related proteins, Arpp19 and Ensa, transforming them into potent interactors and inhibitors of PP2A-B55 [4]. Upon Cdk1 inactivation at the end of mitosis, Gwl activity downregulation allowed PP2A to dephosphorylate Arpp19 and Ensa and to autoactivate [4, 5, 6]. An important PP2A-B55 substrate is the Protein Required for Cytokinesis (PRC) 1, that is dephosphorylated at the end of mitosis at threonine 481 (pT481-PRC1), among several other Cdk1 substrates that are recognized by a commercially available anti phosphorylated Cdk1-substrate (Cdk1 p-sub) [7]. PP2A-B56, instead, has been involved in the mechanisms of spindle assembly and SAC signaling [8]. Indeed, PP2A-B56 has been found to form complex with the SAC protein BubR1 and to localize at kinetochores, proteinaceous centrosomal structures deputed to interact with spindle microtubules [8–12]. Moreover, siRNAs-mediated downregulation of B56 and other PP2A holoenzyme components have been shown to delay spindle assembly, SAC silencing and Cdk1 inactivation [3, 12]. Recently, by using phosphatase inhibitors that target also PP2A, evidence has been provided for a role for protein phosphatases in SAC maintenance [13]. These observations have led to infer that PP2A-B56 activity might affect the SAC-dependent control of Cdk1, however, no direct evidence has been provided for this requirement. In addition, early dephosphorylation of a Cdk1-dependent phosphorylation substrate biosensor appeared not to be affected by the phosphatase inhibitor okadaic acid (OA) at doses that inhibited PP2A activity [14]. Thus, we set out to ask whether activity of PP2A was directly required for the SAC-dependent control of Cdk1 in human cells and gathered evidence suggesting that it is dispensable for this control, thus, PP2A may contribute to spindle assembly and SAC control by structural, rather than catalytic, means.

## RESULTS

**Figure 1:**
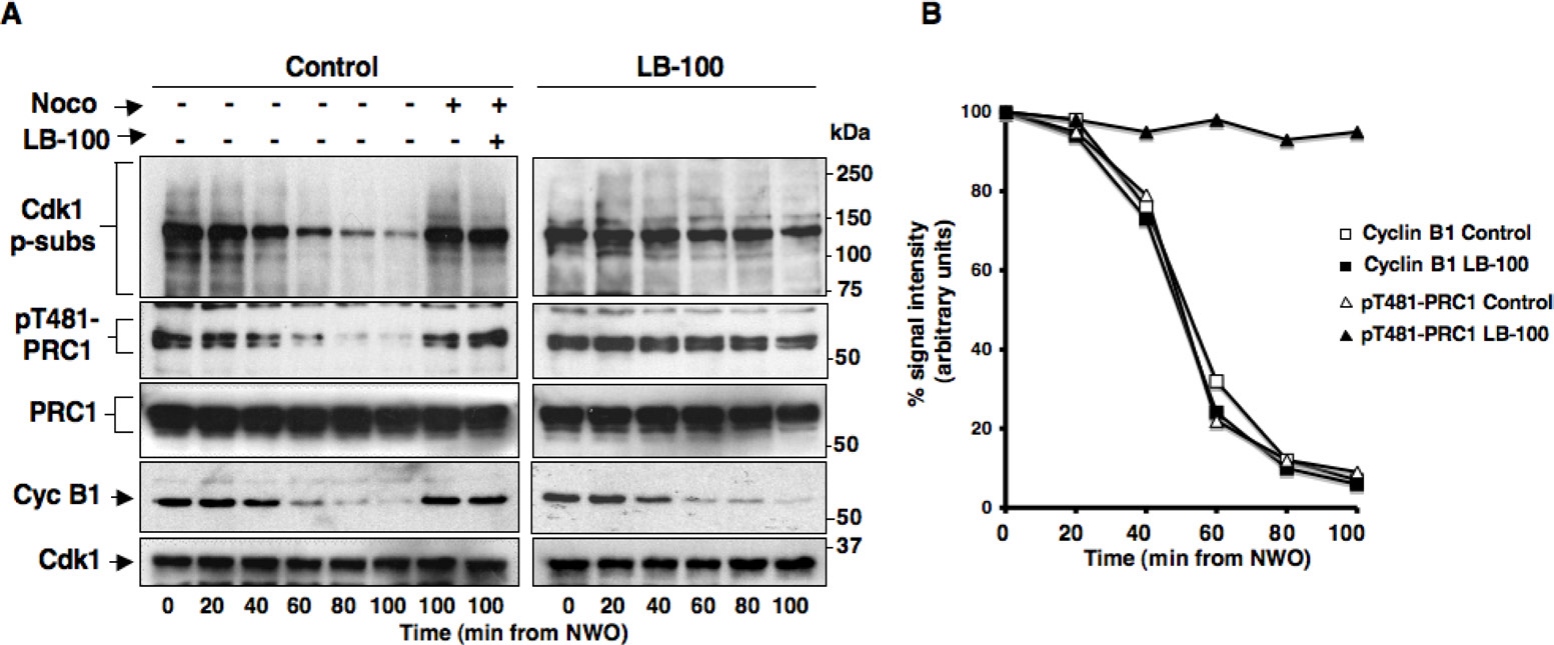

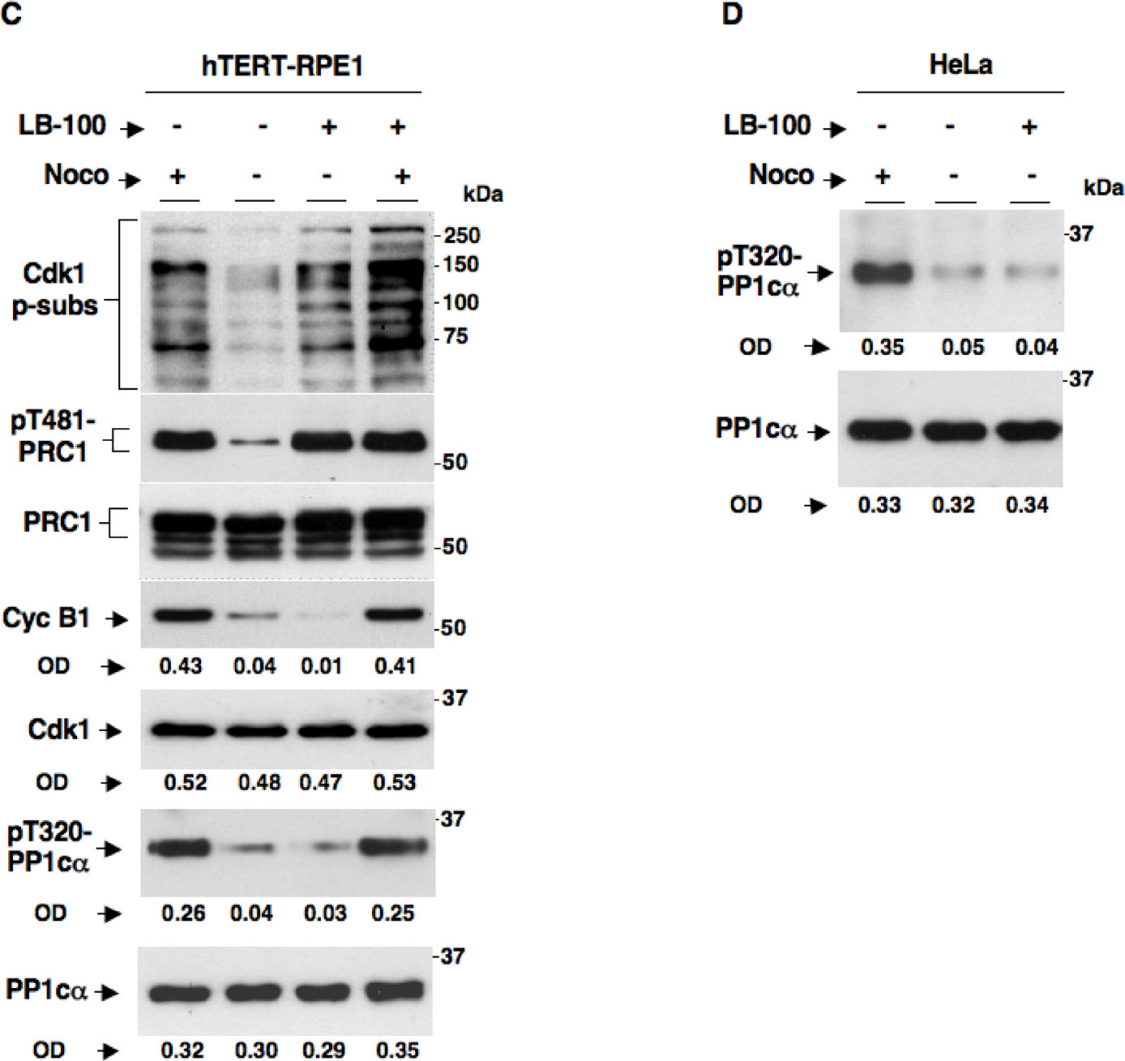
Effects of the PP2A inhibitor LB-100 on SAC maintenance and resolution in HeLa and hTERT-RPE1 cells Nocodazole-treated, prometaphase-arrested, HeLa cells were collected. Upon nocodazole wash out, cells were divided into two sets, one received vehicle (DMSO) as control and the other LB-100 (10 µM). Cells were then taken at the indicated time points of further incubation. Two cell samples in the control set also received again nocodazole (Noco) and one of them LB-100 (LB-100) and, then, were taken at the indicated time points of further incubation. (**A**) Total samples were separated on SDS/PAGE and immunoblotted for phosphorylated Cdk1 substrates (Cdk1 p-subs), pT481-PRC1, PRC1, cyclin B1 (Cyc B1) and Cdk1. (**B**) Optical pT481-PRC1 (triangles) and cyclin B1 (squares) signal density (arbitrary units; normalized for total PRC1 and Cdk1 optical density values, respectively) were plotted as percent of time 0 samples from control (open symbols) and LB-100-treated (filled symbols) cells. (**C**) Nocodazole-treated, prometaphase-arrested, hTERT-RPE1 cells were collected. Upon nocodazole wash out, cells were further incubated for 90 min in: fresh medium plus nocodazole (Noco+), just fresh medium (Noco−), fresh medium plus LB-100 (Noco– LB-100+) or fresh medium plus nocodazole and LB-100 (Noco+ LB-100+). Total samples were separated on SDS/PAGE and immunoblotted for the indicated antigens. OD, optical density values of above signals (arbitrary units). (**D**) Nocodazole-treated, prometaphase-arrested, HeLa cells were collected. Upon nocodazole wash out, cells were further incubated for 90 min in: fresh medium plus nocodazole (Noco+), just fresh medium (Noco−), fresh medium plus 10 µM LB-100 (Noco– LB-100+). Total samples were separated on SDS/PAGE and immunoblotted for the indicated antigens. OD, optical density values of above signals (arbitrary units). The data shown are representative of four independent experiments performed under identical conditions and giving similar results.

To study the possible effects of PP2A catalytic activity inhibition on SAC-dependent control of Cdk1, we set out to determine the effects of LB-100, a rather selective PP2A competitive inhibitor on maintenance and resolution of SAC-dependent mitotic arrest in HeLa cells [15]. Cells were first arrested at prometaphase by a double thymidine block followed by release into fresh medium containing nocodazole, a reversible tubulin polymerization inhibitor, for 12 hours. Detached mitotic HeLa cells were harvested, released from prometaphase arrest into fresh medium and immediately divided into two sets, one received vehicle (dimethyl sulfoxide; DMSO) as control and the other LB-100 (10 µM). Cells were then taken at the indicated time points of further incubation (Figure 1). Two cell samples of the control set were also treated again with nocodazole (Control; Noco+) to keep SAC active, or nocodazole plus LB-100 (Control; Noco+ LB-100+) to keep SAC active and inhibit PP2A, respectively, from time 0 of the experiment and then taken at the indicated time points of further incubation (Figure 1A). Cell lysates were probed for cyclin B1 (Cyc B1) and Cdk1 to monitor Cdk1 inactivation kinetics during mitosis exit; for phosphorylated Cdk1 substrates (Cdk1 p-subs; with an anti phosphospecific antibody recognizing the sequence P-X-pS-P and pS-P-X-K/R; where X = any residue and pS = phosphorylated Ser), PRC1 and phosphorylated PRC1 T481 (pT481-PRC1) to assess PP2A inhibition. Indeed, both Cdk1 p-subs and pT481-PRC1 signals have been shown to be lost during mitosis exit in PP2A-dependent manner [3, 7]. In control cells, Cdk1 p-subs and pT481-PRC1 dephosphorylation ensued following cyclin B1 degradation as SAC was silenced (Figure 1A, 1B). In cells released into LB-100-containing medium, cyclin B1 was degraded with similar kinetics to control cells, however, dephosphorylation of Cdk1 p-subs and pT481-PRC1 were substantially hampered for the duration of the experiment (Figure 1A, 1B). Indeed, quantization of pT481-PRC1 signal intensity showed a drop of more than 80% during 80 min incubation from nocodazole release in control cells, while the signal remained substantially stable in the same temporal range in cells released in the presence of LB-100 (Figure 1B). Quantization of the cyclin B1 signal intensity confirmed that the protein was degraded with similar kinetics in control and LB-100 treated cells (Figure 1B). In control cells treated with nocodazole or nocodazole plus LB-100 from time 0 (Control; Noco+; Noco+ LB-100+), phosphorylated protein levels and cyclin B1 abundance remained stable after 100 min incubation, indicating that LB-100 did not induce SAC override and suggesting that PP2A activity is not required for SAC maintenance (Figure 1A). Together these data indicated that treatment of HeLa cells with the PP2A inhibitor LB-100 substantially inhibited PP2A-dependent dephosphorylations observed during mitosis exit but did not affect cyclin B1 degradation, suggesting that PP2A activity is dispensable for the resolution of SAC-dependent inhibition of Cdk1 inactivation. In addition, LB-100 treatment of HeLa cells under SAC conditions did not override the block to cyclin B1 degradation, suggesting that PP2A is also dispensable for maintenance of SAC-dependent mitotic arrest.

We next asked whether the lack of effects of LB-100 on SAC control observed in HeLa cells was also replicable in non-transformed cells. To this end, we used non-transformed, telomerase-immortalized, human retinal epithelium cells hTERT-RPE1. hTERT-RPE1 cells were prometaphase arrested with nocodazole following a similar protocol to that used for HeLa cells. Upon nocodazole wash out, cells were further incubated for 90 min in: fresh medium plus nocodazole (Noco+), just fresh medium (Noco−), fresh medium plus LB-100 (Noco− LB-100+), or fresh medium plus nocodazole and LB-100 (Noco+ LB-100+). Total samples were separated on SDS/PAGE and immunoblotted for Cdk1 p-subs, pT481-PRC1, PRC1, Cyc B1, Cdk1, PP1 catalytic subunit phosphorylated at threonine 320 (pT320-PP1cα) and PP1 catalytic subunit a (PP1cα) (Figure 1C). Release of hTERT-RPE1 cells from nocodazole arrest resulted in cyclin B1 degradation and Cdk1 p-subs and pT481-PRC1 dephosphorylations after further 90 min incubation in fresh medium (Noco−), while release of these cells into LB-100-containing medium blocked Cdk1 p-subs and pT481-PRC1 dephosphorylation without affecting cyclin B1 degradation (Noco− and Noco− LB-100+; Figure 1C), much like what observed in HeLa cells under similar conditions (Figure 1A). In addition, LB-100 did not induce cyclin B1 degradation in the presence of nocodazole (Noco+ LB-100+), indicating that PP2A inhibition did not cause override of the SAC-dependent arrest (Figure 1C). To determine whether LB-100 was affecting PP1 activity, we analyzed the dephosphorylation of PP1cα at threonine 320 (pT320-PP1cα), a Cdk1-dependent inhibitory site that PP1 autodephosphorylates upon Cdk1 inactivation, and found that it was dephosphorylated as in control cells indicating that LB-100 did not inhibit PP1 catalytic activity (Figure 1C; the lack of effect of LB-100 on pT320-PP1cα dephosphorylation at mitosis exit was also confirmed in HeLa cells, Figure 1D). Thus, also in non-transformed hTERT-RPE1 cells, LB-100 strongly inhibited PP2A-dependent dephosphorylation without affecting cyclin B1 degradation upon SAC inactivation and appeared as well unable to override the SAC-dependent block to cyclin B1 degradation (Figure 1C).

**Figure 2:**
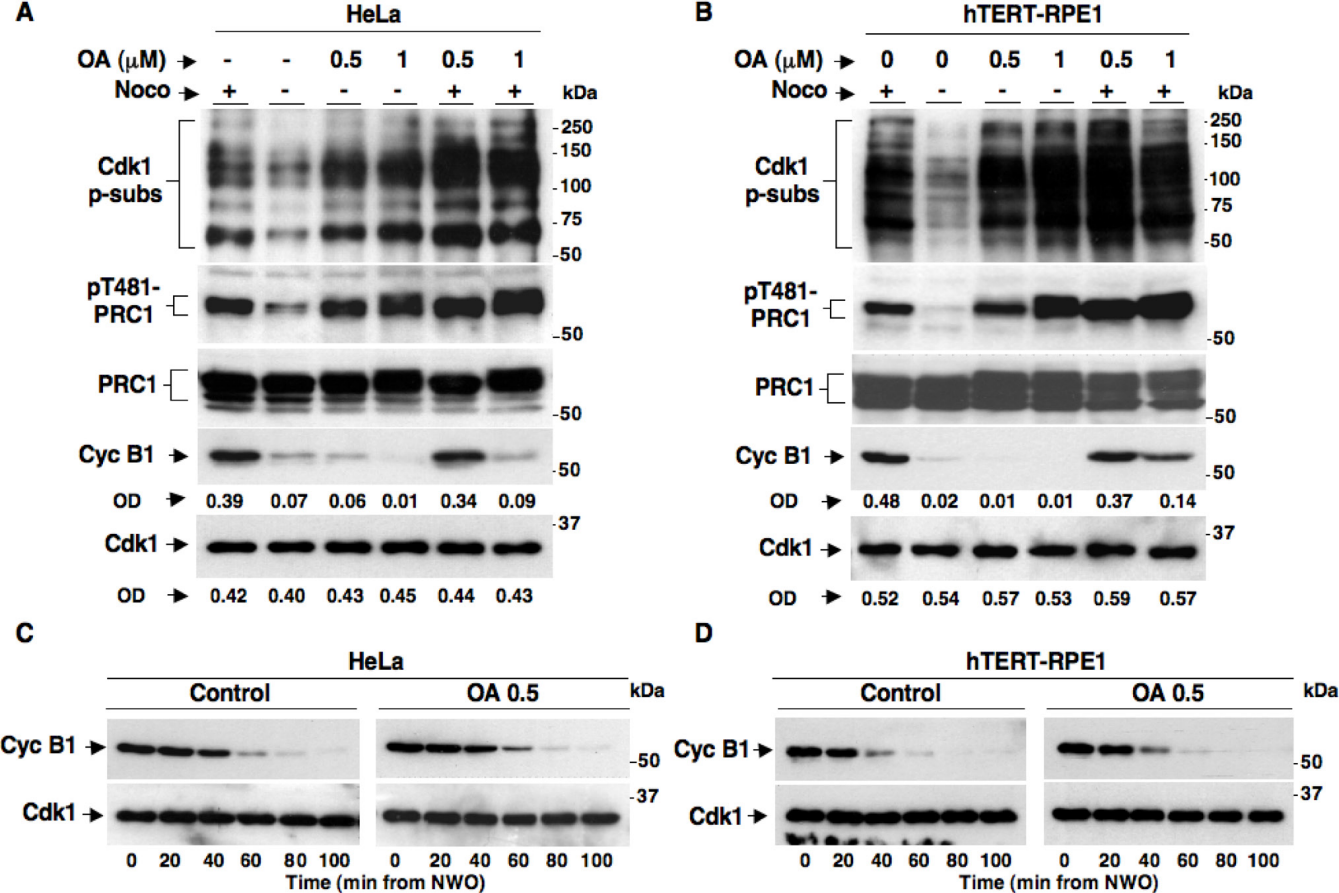
SAC maintenance and resolution in HeLa and hTERT-RPE1 cells treated with the phosphatase inhibitor Okadaic Acid Nocodazole-treated, prometaphase-arrested, (**A**) HeLa and (**B**) hTERT-RPE1 cells were collected. Upon nocodazole wash out cells were further incubated for 90 min in: fresh medium plus nocodazole (Noco+), just fresh medium (Noco−), fresh medium plus OA at 0.5 µM (Noco− OA 0.5), fresh medium plus OA at 1 µM (Noco− OA 1), fresh medium plus nocodazole and OA at 0.5 µM (Noco+ OA 0.5) and fresh medium plus nocodazole and OA at 1 µM (Noco+ OA 1). Nocodazole-treated, prometaphase-arrested (**C**) HeLa and (**D**) hTERT-RPE1 cells were collected. Upon nocodazole wash out, cells were divided into two sets, one received vehicle as control (Control) and the other OA at 0.5 µM (OA 0.5). Cells were then taken at the indicated time points of further incubation. Total samples were separated on SDS/PAGE and immunoblotted for the indicated antigens. OD, optical density values of above signals (arbitrary units). The data shown are representative of four independent experiments per type performed under identical conditions and giving similar results.

OA is a potent PP2A inhibitor that can also inhibit other phosphatases, including PP1, when added to cells in culture in a relatively high (1 µM and above) concentration range [16]. Indeed, in a recent report it has been shown that HeLa cells treated with 1 µM OA were unable to maintain the SAC-dependent block to cyclin B1 degradation, indicating that protein phosphatases were involved in SAC control [13]. However, our data using the LB-100 inhibitor indicated that PP2A activity is irrelevant for SAC-dependent control of cyclin B1 degradation and Cdk1 inactivation (Figure 1). To determine whether we could achieve rather selective inhibition of PP2A activity with OA and analyzed if and how this affected SAC inactivation or maintenance, we treated both HeLa (Figure 2A) and hTERT-RPE1 (Figure 2B) cells with two doses of OA under the following conditions. Nocodazole-treated, prometaphase-arrested, HeLa and hTERT-RPE1 cells were washed out of nocodazole and further incubated for 90 min in: fresh medium plus nocodazole (Noco+ OA−), just fresh medium (Noco− OA−), fresh medium plus OA at 0.5 µM (Noco− OA 0.5), fresh medium plus OA at 1 µM (Noco− OA 1), fresh medium plus nocodazole and OA at 0.5 µM (Noco+ OA 0.5) and fresh medium plus nocodazole and OA at 1 µM (Noco+ OA 1). Total samples were separated on SDS/PAGE and immunoblotted for Cdk1 p-subs, pT481-PRC1, PRC1, Cyc B1 and Cdk1 (Figures 2A, B). In both cell lines released from nocodazole and at both concentrations, OA substantially blocked Cdk1 p-subs and pT481-PRC1 dephosphorylation without affecting cyclin B1 degradation (compare Noco− OA− with Noco− OA 0.5 and Noco− OA 1; Figure 2A, 2B). However, cyclin B1 remained stable in the presence of Noco+ OA 0.5, but was destabilized in the presence of Noco+ OA 1 (Figure 2A, 2B). Thus, our data confirmed that high OA doses, presumably inhibiting PP1 and other phosphatases, induced cyclin B1 degradation by overriding the SAC-dependent block to cyclin degradation (Noco+ OA 1; Figure 2A, 2B), as recently proposed [13]. Nevertheless, our data showed that lower concentrations of OA (0.5 µM) substantially blocked PP2A-dependent dephosphorylations but did not affect cyclin B1 degradation upon SAC resolution nor induced cyclin B1 degradation during SAC-dependent arrest (Noco− OA 0.5 and Noco+ OA 0.5; Figure 2A, 2B). At 0.5 µM, OA did not affect cyclin B1 degradation kinetics even when HeLa or hTERT-RPE1 cells were analyzed at short intervals following release from prometaphase arrest (Figure 2C, 2D). Thus, OA at concentrations that inhibit activity of PP2A is unable to override SAC-dependent arrest and does not affect the resolution of SAC-dependent inhibition of cyclin B degradation, further supporting the conclusion that PP2A activity is dispensable for the SAC-dependent control of Cdk1.

**Figure 3:**
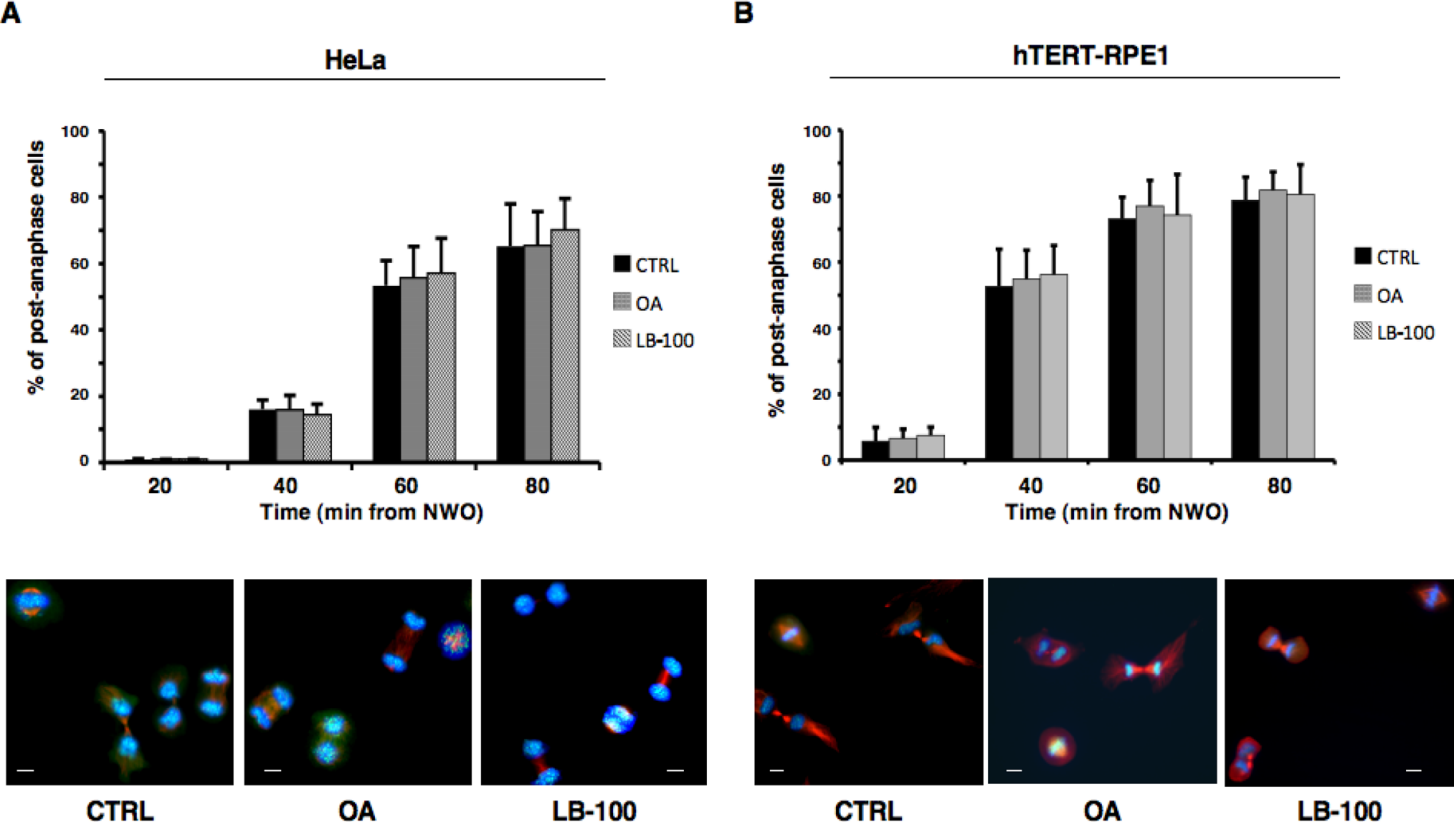
PP2A activity does not substantially affect progression beyond anaphase Nocodazole-treated, prometaphase-arrested, (**A**) HeLa and (**B**) hTERT-RPE1 cells were collected. Upon nocodazole wash out, cells were divided into three sets that received either vehicle as control (CTRL), OA at 0.5 µM (OA), or LB-100. Cells were then taken at the indicated time points of further incubation and spun onto microscopy slides and processed for immunofluorescence staining for α-tubulin and the centromere marker CREST, DNA was stained with Hoechst. Upper graphs: post-anaphase cells were visually scored through microscopy. Error bars refer to variation within three independent experiments performed under identical conditions. Lower photographs: indicative images of HeLa and hTERT-RPE1 cells taken at 60 or 40 min, respectively, of incubation. Scale bars, 10 μm.

The progress beyond anaphase upon release from prometaphase arrest of HeLa and hTERT-RPE1 did not show substantial kinetic difference when analyzed in cells released into vehicle-containing fresh medium, as control, or into fresh medium containing OA (0.5 µM) or LB-100, indicating that resolution of the SAC-dependent block to anaphase onset was independent of PP2A activity (Figure 3A, 3B). At later time points from release, an increased number of binucleated cells could be detected in both cell types treated with phosphatase inhibitors (not shown), indicative of a requirement for PP2A activity in cytokinesis as previously suggested [3, 4, 7].

**Figure 4:**
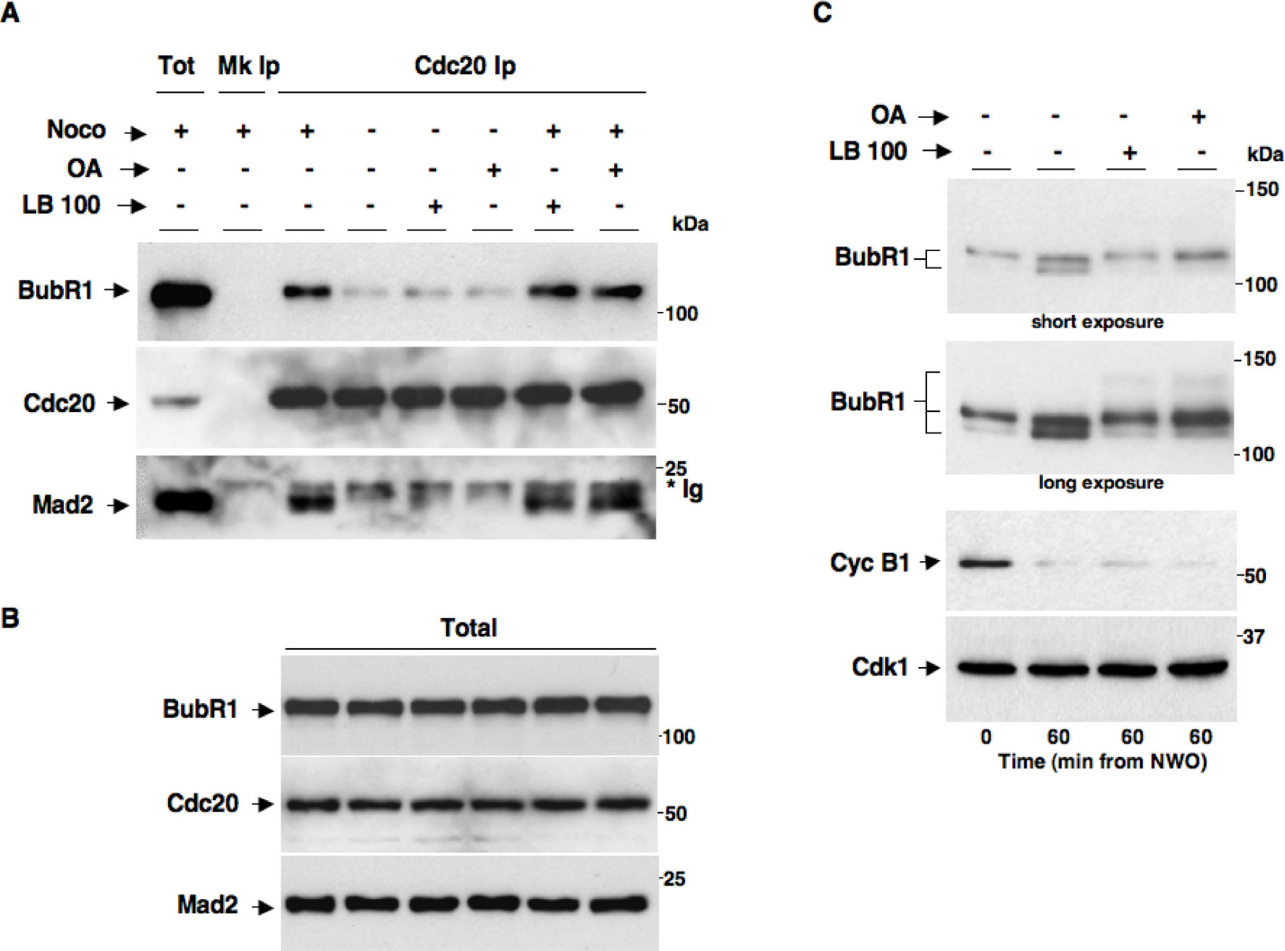
PP2A activity does not affect MCC maintenance and disassembly Nocodazole-treated, prometaphase-arrested, HeLa cells were washed out of nocodazole and further incubated for 60 min in: fresh medium plus nocodazole (Noco+), just fresh medium (Noco−), fresh medium plus LB-100 (10 µM; Noco− LB-100+), fresh medium plus OA (0.5 µM; Noco− OA+), fresh medium plus nocodazole and LB-100 (10 µM; Noco+ LB-100+) and fresh medium plus nocodazole and OA (0.5 µM; Noco+ OA+). Cdc20 was immunoprecipitated (Ip) from cell lysates. (**A**) Cdc20 Ips (Cdc20 Ip) were resolved on SDS/PAGE and subsequently probed by immunoblotting for the indicated antigens (a total cell lysate, Tot; and mock Ip, Mk Ip, were also included as control in the SDS/PAGE; the asterisk marks immunoglobulin signal, *Ig). (**B**) Total lysates were also probed for the indicated antigens. (**C**) Nocodazole-treated, prometaphase-arrested, HeLa cells were washed out of nocodazole and further incubated for 60 min in: fresh medium (LB-100- OA−), fresh medium plus LB-100 (10 µM; LB-100+ OA−) or fresh medium plus OA (0.5 µM; LB-100- OA+). BubR1 was probed on blots of long, higher resolving, SDS/PAGE runs of total lysates (short and long blot exposures are shown). Total lysates were also probed for the other indicated antigens. The data shown are representative of three independent experiments performed under identical conditions and giving similar results.

At the end of mitosis, Cdk1 inactivation and anaphase onset are determined by activation of the ubiquitin ligase Anaphase-Promoting Complex/Cyclosome (APC/C) that promotes cyclin and securin, an anaphase inhibitor, proteolysis in association with its coactivator Cdc20. The SAC delays anaphase onset and Cdk1 inactivation until mitotic spindle assembly by stimulating formation of a mitotic checkpoint complex (MCC) in which Mad2 and BubR1, two major SAC effector proteins, bind Cdc20 and restrain APC/C^Cdc20^ activation. We, thus, set out to determine whether PP2A activity affected MCC during SAC control by coimmunoprecipitation (coIp) experiments. Nocodazole-treated, prometaphase-arrested, HeLa cells were washed out of nocodazole and further incubated for 60 min in: fresh medium plus nocodazole (Noco+), just fresh medium (Noco−), fresh medium plus LB-100 (Noco− LB-100+), fresh medium plus OA (0.5 µM; Noco− OA+), fresh medium plus nocodazole and LB-100 (Noco+ LB-100+) and fresh medium plus nocodazole and OA (0.5 µM; Noco+ OA+; Figure 4). Cdc20 coIps from cell lysates were probed for BubR1, Cdc20 and Mad2 (Figure 4A). Total lysates were also probed for BubR1, Cdc20 and Mad2 (Figure 4B). While in SAC-arrested cells (Noco+) significant amounts of Mad2 and BubR1 were found bound to Cdc20, marking an assembled MCC, in cells released from nocodazole (Noco−), thus allowed to assemble spindles and inactivate SAC, Mad2 and BubR1 substantially dissociated from Cdc20, marking MCC disassembly, regardless of the presence of LB-100 or OA (Noco− LB-100+ and Noco− OA+; Figure 4A). These data indicate that PP2A activity has no direct role in SAC resolution and MCC disassembly. In addition, PP2A does not appear required for SAC-dependent Cdk1 stabilization since the MCC remained stable and no SAC override was observed when PP2A was inhibited in the presence of nocodazole (compare Noco+ with Noco+ LB-100+ or Noco+ OA+; Figure 4A). These data are also in agreement with recently published observations indicating that the MCC amount and stability were not significantly affected in PP2A-B56-depleted cells, although the phosphorylation status of BubR1, analysed by migration shift on SDS/PAGE, was indeed affected by PP2A-B56 depletion [17]. Thus, we analysed the migration pattern of BubR1 from HeLa cells exiting mitosis in the absence or presence of LB-100 or OA by long, higher resolving, SDS/PAGE runs (Figure 4C). HeLa cells were washed out of nocodazole and further incubated for 60 min in: fresh medium (LB-100- OA−), fresh medium plus LB-100 (LB-100+ OA−) or fresh medium plus OA (0.5 µM; LB-100- OA+; Figure 4C). Indeed, BubR1 from cells released into fresh medium resolved into faster migrating forms compared to mitotic cells at time 0, indicative of dephosphorylation, while BubR1 from cells released into LB-100- or OA−containing medium resolved in slow migrating forms, similar to that from mitotic time 0 cells, and even slower migrating forms indicative of hyperphosphorylation (Figure 4C). Despite lack of BubR1 dephosphorylation, cyclin B1 degradation proceeded similarly in all samples (Figure 4C). These data suggest that also PP2A-B56 activity in particular is efficiently inhibited under our conditions but this does not affect Cdk1 inactivation.

**Figure 5:**
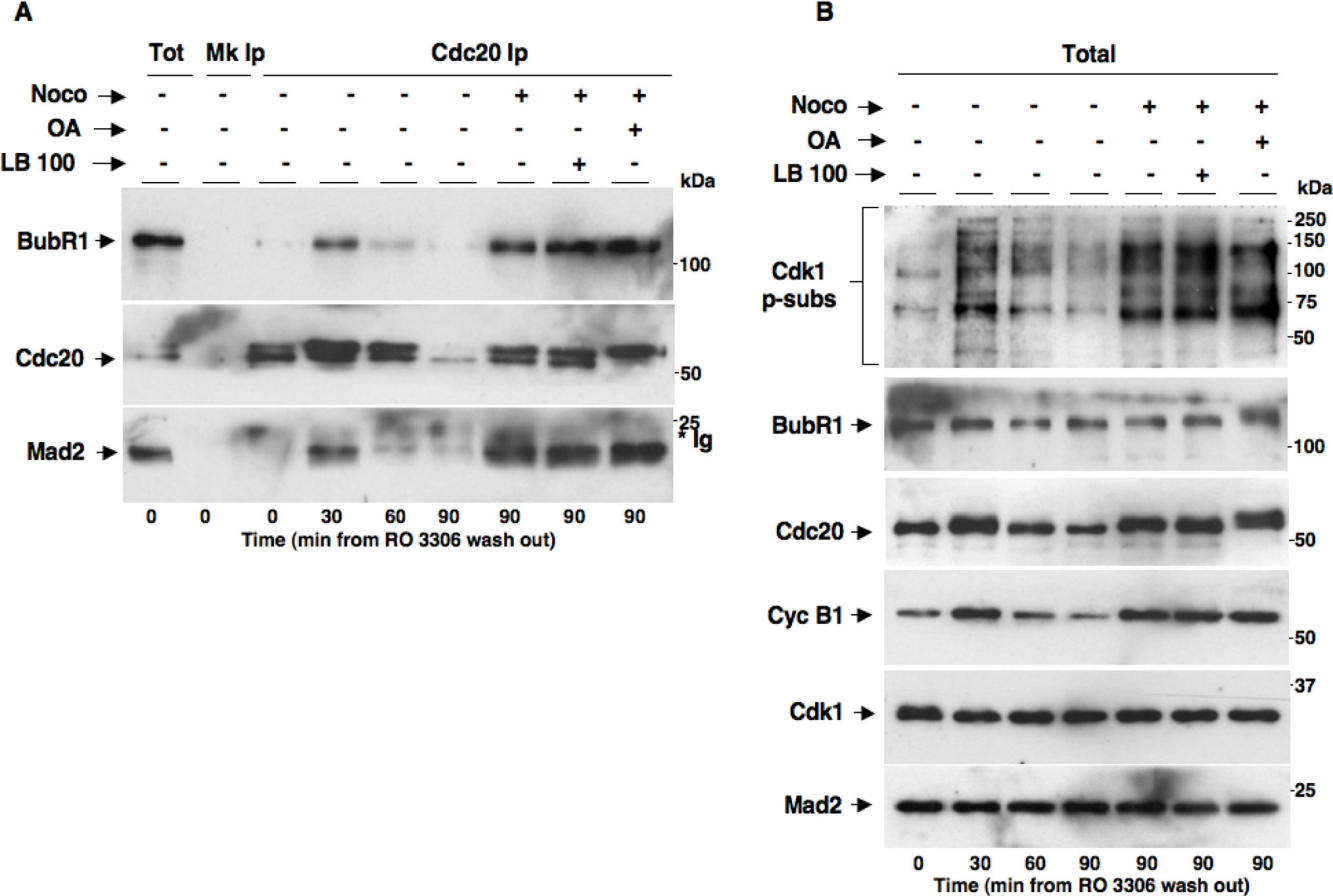
PP2A activity does not affect MCC formation and implementation of SAC-dependent mitotic arrest HeLa cells were treated for 18 hours with RO3306 (10 µM). Upon RO3306 wash out, cells were released into: fresh medium (Noco− OA− LB-100-), fresh medium plus nocodazole (Noco+ OA− LB-100-), fresh medium plus nocodazole and LB-100 (Noco+ OA− LB-100+) and fresh medium plus nocodazole and OA (Noco+ OA+ LB-100-). Cell samples were taken at the indicated time points of further incubation and (**A**) Cdc20 Ips (Cdc20 Ip) were resolved on SDS/PAGE and subsequently probed by immunoblotting for the indicated antigens (a total cell lysate, Tot; and mock Ip, Mk Ip, were also included as control in the SDS/PAGE; the asterisk marks immunoglobulin signal, *Ig). (**B**) Total lysates were also probed for the indicated antigens. The data shown are representative of four independent experiments performed under identical conditions and giving similar results.

We asked whether PP2A activity was required for MCC formation and implementation of the SAC effector branch [18, 19]. To this end, we synchronized HeLa cells in G2 by a 18-hour treatment with the Cdk1 inhibitor RO3306. From RO3306-dependent G2 arrest, cells were released into fresh medium and sampled at various time points of further incubation or released into nocodazole-containing medium in the absence (Noco+ LB-100– OA−) or presence of LB-100 (10 µM, Noco+ LB-100+) or OA at 0.5 µM (Noco+ OA+) and sampled after further 90 min of incubation (Figure 5). Cdc20 coIps from cell lysates were probed for BubR1, Cdc20 and Mad2 (Figure 5A). Total lysates were also probed for Cdk1 p-subs, BubR1, Cdc20, Cyc B1, Cdk1 and Mad2 (Figure 5B). In cells released just into fresh medium, BubR1 and Mad2 transiently bound to Cdc20, MCC reached a maximum by 30 min while they completely dissociated by 90 min of incubation (Figure 5A). In cells released into nocodazole-containing medium, instead, MCC was stabilized by 90 min of incubation as high amounts of BubR1 and Mad2 were still associated with Cdc20 (Figure 5A) and the cells remained in mitosis as indicated by high Cdk1 p-subs and cyclin B1 signals (Figure 5B), as a consequence of an activated SAC. Importantly, nocodazole-dependent MCC stabilization and mitotic arrest were not affected by the presence of either LB-100 or OA, indicating that PP2A was dispensable also for the implementation of a functional SAC.

Together our findings indicate that PP2A activity is neither absolutely required for MCC formation and SAC activation and maintenance nor for MCC disassembly and SAC silencing.

## DISCUSSION

The SAC is a safeguard mechanism evolved to ensure accurate chromosome segregation; this occurs by inhibiting the APC/C until all the chromosomes reach bipolar attachment to spindle microtubules [20]. Spindle assembly and SAC control rely on protein phosphorylation and several kinases have been involved in these mechanisms [8, 20]. On the other hand, protein phosphatases play essential roles in homeostatically regulating these processes [8, 19, 20, 21, 22]. In original works in yeast, PP1 activity was described to have a crucial role in silencing the SAC upon spindle assembly [23, 24]. PP2A-B55 activity has been shown to be required for mitosis completion downstream Cdk1 inactivation and to be necessary to control late mitotic events like cytokinesis and reformation of the interphase nucleus [4, 5, 6, 7]. PP2A-B56, instead, has been involved in chromosome alignment at metaphase and SAC control. Indeed, the SAC effector protein BubR1 has been shown to physically interact with the B56 subunit while small interfering RNAs-mediated B56 downregulation has been shown to affect chromosome alignment and segregation, leading to the hypothesis that PP2A-B56 activity was required to antagonize Mps1 and Aurora B kinases, promote stable kinetochore-microtubule interactions and silence the SAC [9, 10, 11]. However, other observations suggested that PP2A-B56 was required for chromosome congression at the metaphase plate, rather than to stabilize kinetochore-microtubule interactions by antagonizing Aurora B action on kinetochore proteins [25]. Yet another study indicated that PP2A-B56 could be required to recruit PP1 at kinetochores, and that the activity of PP1 would ultimately be required to silence the SAC [12]. Nevertheless, despite all these studies, a direct role for PP2A phosphatase activity in SAC regulation has been inferred but never directly demonstrated [25, 26]. In addition, OA did not prevent early dephosphorylation of a Cdk1-dependent phosphorylation biosensor during mitosis exit at doses that inhibited PP2A activity [14].

In this work we asked whether PP2A catalytic activity was directly involved in SAC and MCC-dependent control of Cdk1. To this end, we achieved significant PP2A inhibition by using the chemical inhibitors LB-100 and OA at doses that selectively inhibited PP2A activity. In cells released from a SAC-dependent mitotic arrest, these inhibitors substantially inhibited PP2A-dependent dephosphorylations that mark mitosis exit but did not block silencing of the SAC effects on Cdk1 stabilization as demonstrated by the fact that disassembly of the MCC and cyclin B1 degradation were unaffected by these inhibitors (Figures 1, 2, 3 and 4). In addition, cells mounted and maintained a rather consistent SAC-dependent mitotic arrest when incubated in the presence of either PP2A inhibitor plus nocodazole (Figure 5). Thus, we concluded that the catalytic activity of PP2A is dispensable for activation, maintenance and silencing of the SAC-dependent control of Cdk1. Together with the previous observations obtained through genetic downregulation of PP2A subunits, our data suggest a possible role for PP2A in SAC control independently of its catalytic activity. In this perspective the PP2A holoenzyme, likely with B56 as B subunit, could be envisaged as the basis of multiprotein hubs required to control chromosome movements or to physically link specific structures, like kinetochores, to signaling molecules, like SAC proteins, for spindle assembly and SAC control in a catalytic activity-independent fashion. Alternatively, the potential requirement for PP2A-B56 catalytic activity in spindle assembly does not affect the control of MCC assembly-disassembly and SAC-dependent control of Cdk1. Finally, while PP2A inhibition may help cancer therapy in combination with DNA damaging therapeutics by promoting aberrant progression into mitosis, our results cast doubt on the possibility that targeting PP2A activity may help to improve efficacy of widely used anticancer drugs that target the mitotic spindle like taxanes and vinca alkaloids by affecting the SAC-dependent mitotic delay [27, 28].

## MATERIALS AND METHODS

### Cell cultures and treatments

HeLa cells were grown in Dulbecco’s Modified Eagle’s Medium (DMEM; Sigma-Aldrich, St. Louis, MO, USA), hTERT-RPE1 cells were grown in Dulbecco’s Modified Eagle’s Medium/Nutrient Mixture F-12 (DMEM/F12; Thermo Fischer Scientific, Waltham, MA, USA), both supplemented with 10% fetal bovine serum (FBS; GE Healthcare Hyclone, Little Chalfont, Buckinghamshire, UK) and penicillin–streptomycin (Sigma-Aldrich, St. Louis, MO, USA) as described [29]. Prometaphase-arrested cells were obtained by a double thymidine (4 mM; Sigma-Aldrich, St. Louis, MO, USA) block (18 hours each, separated by a 6 hours incubation in fresh medium) followed by release into fresh medium containing nocodazole (500 nM; Calbiochem, Billerica, MA, USA). Cells were further incubated for 12 or 14 hours for HeLa and hTERT-RPE1, respectively. Then, cells that detached from substrate were collected and released from prometaphase-arrest by two washes with phosphate-buffer saline (PBS; EuroClone, Milan, IT) followed by two washes with fresh medium and final incubation in fresh medium. G2-arrested HeLa cells were obtained by one thymidine block followed by release into fresh medium containing RO3306 (10 μM; Calbiochem, Billerica, MA, USA) for 17 hours. Cells were released from the G2-arrest by two washes with PBS followed by two washes with fresh medium and final incubation in fresh medium. Cells released from either prometaphase- or G2-arrest, were collected at indicated time points of further incubation and lysed in NP-40/EB lysis buffer (0.2% NP-40; 80 mM 2-glycerophosphate, 10 mM MgCl_2_ and 20 mM EGTA; Sigma-Aldrich, St. Louis, MO, USA). Lysates were incubated 30 minutes on ice and spun for 20 minutes at 13.200 rpm, in a refrigerated centrifuge. Synchronized cells were treated with okadaic acid (Calbiochem, Billerica, MA, USA) at the indicated final concentrations or LB-100 (Selleckchem, Houston, TX, USA) at 10 µM.

### Antibodies and immunological techniques

Primary antibodies used for immunoblots detection: rabbit polyclonal anti-cyclin B1 (1:1000), mouse monoclonal anti-CDC2 (Cdk1; 1:1000), rabbit polyclonal anti-PRC1 (1:250), mouse monoclonal anti-p-T481-PRC1 (1:300), mouse monoclonal anti-Mad2 (1:300), rabbit polyclonal anti-P55 CDC (Cdc20; 1:500), goat polyclonal anti-PP1α (1:200), antibodies were purchased from Santa Cruz Biotechnology (Dallas, TX, USA). Rabbit monoclonal anti-phospho-serine Cdk1 substrates (Cdk1 p-subs; recognizing PXpSP and pSPXK/R; 1:1000) and rabbit polyclonal anti phospho-pT320-PP1α (1:750) antibodies were purchased from Cell Signaling Technology (Danvers, MA, USA) while mouse monoclonal anti-BubR1 (1:1000) antibody was purchased from BD Biosciences (San Jose, CA, USA). Secondary antibodies were peroxidase-conjugated donkey anti-rabbit (1:5000), anti-mouse (1:3000) or anti-goat (1:5000) IgG (GE Healthcare, Milan, IT) and signals were detected using an enhanced chemiluminescence kit (ECL; Cyanagen, Bologna, IT). Immunoblot signal intensity was measured by using ImageJ software (National Institutes of Health, Bethesda, MD, USA). For immunoprecipitation (Ips) experiments, cell lysates were incubated 30 minutes on ice and spun 3× for 20 minutes at 13.200 rpm, in a refrigerated microfuge. Cdc20 Ips were performed using agarose-conjugated anti p55 mouse monoclonal IgG (Santa Cruz Biotechnology, Dallas, TX, USA). Ips were incubated on a rotator wheel over night at 4°C and washed three times with NP-40/EB. Total lysate samples and Ips were separated on SDS/PAGE and blotted onto nitrocellulose membrane (Amersham-GE Healthcare, Milan, IT). For indirect immuno-fluorescence, cells were spun on microscopy slides, washed in PBS, fixed with 3.7% paraformaldehyde (Sigma-Aldrich, St. Louis, MO, USA) in PBS for 10 minutes at room temperature and permeabilized with 0.25% Triton X-100 (Sigma-Aldrich, St. Louis, MO, USA) in PBS for further 10 minutes. After blocking with 3% BSA (Sigma-Aldrich, St. Louis, MO, USA) in PBS for 1 hour, samples were incubated for 3 hours with mouse anti α-tubulin (1:500; Sigma-Aldrich, St. Louis, MO, USA) and human anti CREST (1:100; Fitzgerald Industries International, North Acton, MA, USA) primary antibodies, both in PBS + 1% BSA. After 3 PBS washes, samples were incubated with secondary antibodies (1:300; Jackson ImmunoResearch Laboratories Inc., West Grove, PA, USA) in PBS + 1% BSA for 1 hour at room temperature. DNA was stained by incubation with Hoechst 33258 (10 mg/ml; Santa Cruz Biotechnology, Dallas, TX, USA) in PBS for 10 minutes. Samples were observed and scored for chromosome segregation using an Axiovert 200 M inverted microscope, equipped with the Apotome slider module with a 40× objective (Zeiss, Oberkochen, Germany).
